# New agents for the treatment of lymphoid leukemia and lymphoma: focus on recent FDA approvals

**DOI:** 10.15190/d.2014.6

**Published:** 2014-03-31

**Authors:** Andreea Lucia Stancu, Mitchell R. Smith, Alexandru Almasan

**Affiliations:** Department of Pathology, Beth Israel Deaconess Medical Center and Harvard Medical School, Boston, MA; Department of Hematologic Oncology and Blood Disorders, Cleveland Clinic, OH, USA; Department of Cancer Biology, Lerner Research Institute, Cleveland Clinic, Cleveland, OH, USA

**Keywords:** FDA approved drugs, leukemia, lymphoma, ibrutinib, obinutuzumab, lenalidomide

## Abstract

Leukemia and lymphoma are systemic malignancies that represent half of all childhood cancers, though 90% occur in adults. Various  treatment options are available, but therapy is mainly systemic chemotherapy plus appropriate monoclonal antibodies. In certain situations radiotherapy and bone marrow transplantation play a role. Some types/subtypes of these diseases are potentially curable, yet many leukemias and lymphomas do not properly respond to current therapies. Although the FDA (US Food and Drugs Administration) approvals of new drugs have shown a small increasing trend between 2007-2012, overall, the trend of new approvals remains relatively steady between 2006-2013, with a peak of 39 new drugs approved in 2012 and a drop in the new FDA drug approvals in 2013, to 27. Drugs approved for cancer treatment have shown a similar trend. Between 2006-2013, at least one drug was approved every year for the treatment of particular types of lymphoma or leukemia, except in 2010, with a peak of 5 new approvals in 2012. Between January 2013-March 2014, several important new approvals were made: ibrutinib for the treatment of CLL and mantle cell lymphoma (MCL), obinutuzumab for the treatment of CLL (in combination with chlorambucil), and lenalidomide for the treatment of mantle cell lymphoma. The results, importance, adverse effects and mechanisms of action of these agents are discussed in this review. These results held promise and their discovery and approval for the treatment of CLL and MCL is a major step forward. However, the emergence of resistance and the lack of cures need to be addressed by rational development of combination therapy, as well as development of novel drugs with enhanced potency or different mechanism of action, to achieve better overall and complete response rates with decreased toxicity.

## Introduction

Hematologic malignancies are generally characterized by their hematologic cell of origin, first whether they are lymphoid or myeloid. Lymphoid leukemias and lymphomas are systemic malignancies that represent about half of all childhood cancers^1^, though 90% of leukemias are actually found in adults^2^.

Leukemia (meaning “white blood”: greek words for "white" (leukos) and "blood" (haima))^3^ is a type of blood and bone marrow malignancy, part of a larger group of hematological malignancies. Acute leukemias are characterized by the abnormal proliferation of blasts, which are immature leukocytes (white blood cells), in the bone marrow^4^. Blast cell proliferation leads to a wide variety of symptoms due to decreased production of several types of normal cells in the bone marrow, including: erythrocytes (anemia), platelets (risk of bleeding), and mature leukocytes (risk of infection). Acute leukemias can involve other organs, such as the brain and spinal cord^4,5^. Chronic leukemias generally involve more mature, less proliferative cells than acute variants. Thus, leukemias are classified as: (i) chronic myelogenous leukemia (CML); (ii) chronic lymphocytic leukemia (CLL); (iii) acute myelogenous leukemia (AML); and (iv) acute lymphoblastic leukemia (ALL). In addition to these four main groups of leukemias, there are few other rarer types, such as hairy cell leukemia that arise from mature memory B cells^4,6,7^.

In contrast to leukemias, lymphomas, defined as malignant proliferation of lymphocytes, more typically present as a solid tumor^8^. The separation of lymphoid leukemia and lymphoma is somewhat artificial, given that they arise from the same lineage, and in some cases such as CLL depend solely on an arbitrary definition of white blood cell count. Several types of characteristics are used to classify lymphomas in categories that are important for the individualized treatment and prognosis for each category. Classification of the lymphoma is complex but now generally used is the WHO classification, which was established in 2001 and revised in 2008^9,10^ organizing lymphomas based on the cell types involved in three main groups: T-cell, B-cell and NK (Natural Killer) cell lineages, and further subdivided based on clinical, immunophenotypic and genetic factors.

Importantly, although Hodgkin lymphoma was considered a separate group, it is a tumor of B lymphocytes^9,10^**.**

There are many treatments available for particular types of leukemias and lymphomas. As these are almost always systemic diseases, therapy is generally systemic immunochemotherapy, with radiotherapy and bone marrow transplantation indicated in certain situations^11-18^. Some types/subtypes of lymphoid leukemias and lymphomas, generally the more rapidly growing subtypes, can be curable^8^.

**Figure 1:**
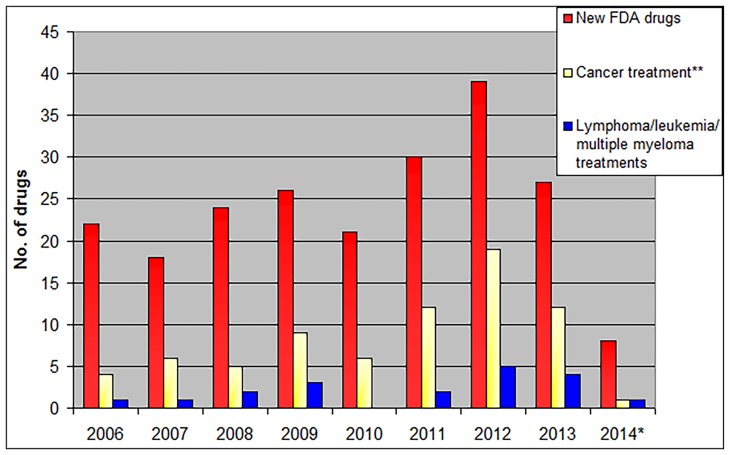
FDA approved drugs between 2006-2014 (January-March) The number of FDA approved drugs showed an increasing trend between 2007-2012, with a maximum of 39 new FDA drugs approved in 2013. The number dropped back to 27 new FDA approved drugs in 2013 (Red); A similar pattern is seen in the case of the FDA approved drugs for the cancer treatment (both new drugs and older, previously approved drugs, that are now approved for a different pathology are considered) (Yellow); At least 1 new FDA drug was approved by FDA for the treatment of leukemia/lymphoma/ multiple myeloma each year between 2006-2014, except in 2010 (Blue)^19,20^; * 2014 (January - March); ** both new approved drugs and older drugs approved for a different type of pathology in that particular year are considered;

In particular, CLL and Mantle Cell Lymphoma (MCL) chemotherapy treatments are started when the patients become symptomatic. A wide variety of treatments are available for the CLL, including alkylating agents, nucleoside analogues and biologics, alone or in combination, and allogeneic stem cell transplantation^21-25^. First line treatment of aggressive and symptomatic MCL in younger patients include rituximab (anti-CD20 antibody)-based combination chemotherapies. Autologous stem cell transplantation follows this treatment. Rituximab in combination with HyperCVAD (cyclophosphamide, vincristine, doxorubicin, dexamethasone alternating with methotrexate and cytarabine) plus rituximab, R-CHOP (rituximab, cyclophosphamide, doxorubicin, vincristine, and prednisone), and rituximab in combination with bendamustine are several other first line therapies^26-32^.

However, the emergence of resistance and the lack of disease cure in many settings need to be addressed by the development of novel and efficient drugs^4,12^.

FDA (US Food and Drugs Administration) approvals of new drugs have shown a slight increasing trend between 2007-2012, with a peak of 39 new drugs approved in 2012. The number of new drugs approvals dropped in 2013 to 27. Overall, between 2006 and 2013, the trend of new approvals remains relatively steady^19,20^. The trend for the new drugs approved in general, new cancer drug approvals, and new drugs approved for the treatment of leukemia & lymphoma by the FDA is shown in **Figure 1**.

Recently (January 2013-March 2014), several important drugs were approved for the treatment of leukemia (CLL) and lymphoma (MCL). These drugs are further discussed in this article (**Table 1**).

**Table 1:** Drugs approved by FDA between January 2013-March 2014 for the treatment of lymphoid lymphoma/leukemia mo = months; ORR = overall response rate; CR = complete response; PR = partial response; Note: information provided by FDA website and other sources - see main text^19-36^

Name and company	Approved for	Results	Adverse effects	Date of approval	FDA expedited review and approval pathway
Ibrutinib (Imbruvica), Pharmacyclics	Previously treated MCL patients / Previously treated CLL patients	ORR of 68%; CR rate 21%+ PR rate 47% / ORR of 58%	trombocytopenia, neutropenia, anemia, diarrhea, constipation pain (muscles, bones and joints), rash, fever, nausea, edema, stomatitis, sinusitis, dizziness	*2013.11; Approved in only 4.5 mo. / 2014.02;*	*Fast track; Breakthrough, Priority Review and accelerated approval* (for previously treated MCL patients)
Obinutuzumab (Gazyva) Genentech	First-line CLL treatment (+ chlorambucil)	ORR ~ 75% (with chlorambucil), vs. chlorambucil alone ORR 32%.	risk for infusion reactions, leucopenia, thrombocytopenia, anemia, Progressive Multifocal Leuko-encephalopathy (PML), Hepatitis B Virus (HBV) reactivation, fever, pain (muscle and joints), cough	*2013.10; Approved in only 6.3 mo.*	*Breakthrough, Priority Review*
Lenalidomide (Revlimid) Celgene	MCL patients	ORR 26%; CR 7%	leucopenia/neutropenia, thrombocytopenia, anemia, diarrhea/constipation, nausea, cough, fever, rash/pruritus, dyspnea, peripheral edema	*2013.06;*	

## Ibrutinib (Imbruvica) – a novel FDA approved drug for the treatment of CLL (2014) and MCL (2013)

Ibrutinib (Imbruvica) is a new drug produced by Pharmacyclics (Sunnyvale, California, USA) that was recently approved in a short period of time by FDA in November 2013 as single agent therapy of patients with previously treated MCL. Ibrutinib is a Bruton’s Tyrosine Kinase (BTK) inhibitor that demonstrated a response rate of 68% at 560 mg/day of orally-administered ibrutinib. A complete response to ibrutinib therapy was observed in 21% and a partial response in 47% of the treated patients^19-21^. The median time to complete response was 5.5 months (1.9 months to response), with a median response duration of 17.5 months and median progression-free survival of almost 14 months^19,20,22^.

Based on these encouraging results, it was one of the first drugs that received the FDA’s “Breakthrough” approval(designation introduced in July 2012)^19,20,22^. Only 3 of the 27 newly approved drugs in 2013 received the designation of “Breakthrough” therapy, which means that ibrutinib may have improved clinical endpoints compared to existing therapies^19,20,22^. Thus, ibrutinib was approved in only 4.5 months^19,20^, which makes it one of the fastest FDA approvals in recent years. Ibrutinib is also the only new FDA approved drug in 2013 to receive all four designated expedited review and approval pathways: “Fast track”, “Accelerated approval” “Breakthrough” and “Priority Review”^19,20^.

Noteworthy, in February 2014, FDA extended the approved indication for ibrutinib to treatment of CLL patients who have already received other treatments^23^***. ***Showing an overall response rate of 58% and a duration of response between 5.6 to 24.2 months with a dose of 420 mg administered orally, until progression of the disease or unacceptable toxicity occurred, Ibrutinib received an accelerated FDA approval. It is too early to assess any effect on survival^23^***.***

**Ibrutinib has several observed side effects, including thrombocytopenia (increased risk of bleeding or bruising), neutropenia (increased risk of infections), anemia (increased fatigue, dyspnea), diarrhea/constipation, arthralgias/myalgias, rash, fever, nausea, edema, stomatitis, sinusitis and dizziness^2,19,20^***.***

**BTK is a critical kinase found downstream of the B-Cell Receptor (BCR) signal transduction pathway. Compared to other BTK inhibitors, such as dasatinib, ibrutinib has higher specificity for binding BTK, since it covalently and irreversibly binds to BTK, and is less inhibitory to other targets^22^. In fact, it represents one of the first irreversible kinase inhibitors used in the clinic. Its major mechanisms of action in MCL and CLL are both through a direct on target effect on the BTK (inhibiting cell survival and proliferation of cancer cells) and an indirect effect through the stromal cells that interact with the cancer cells^22^. Ibrutinib mobilizes cells resulting in increase of the MCL and CLL cells in the blood that may last for months. This can confound the interpretation of response, especially in CLL where the blood lymphocyte count is a main factor in assessing response^22,24^.

**Although very promising, ibrutinib therapy is also very expensive. At ~ $ 92 each pill, the estimated treatment cost is of $130,000/year, which makes it one of the most expensive drugs used for cancer treatment^22,24^, when one considers that the goal is long-term therapy.This certainly has the potential to limit its use, and to curtail the ability to develop combination therapies. **

## Obinutuzumab (Gazyva) – a novel FDA approved drug for the therapy of previously untreated CLL patients (2013)

Obinutuzumab (Gazyva) is a novel drug produced by Genentech that was approved by FDA in October 2013 in only 6.3 months as a first line therapy of previously untreated CLL patients in combination with chlorambucil^26,27^. Obinutuzumab is a humanized IgG1 type II anti-CD20 monoclonal antibody^26^. Though CLL is a B cell leukemia of older patients (median age at diagnosis ~ 70 years), many clinical trials include a younger patient population. In a randomized phase 3 trial in older, less fit patients with CLL the overall response rate of ~ 75% for obinutuzumab in combination with chlorambucil was significantly better than that of chlorambucil alone (ORR 32%), and also of rituximab + chlorambucil (65%). The primary endpoint if the trial was progression-free survival (PFS), and for this obinutuzumab + chlorambucil was 27 months, chlorambucil alone about 12 months and rituximab + chlorambucil 15 months^27^. The FDA approved obinutuzumab based only on the comparison with chlorambucil alone.

Based on its results obtained in clinical trials, obinutuzumab* is the first drug *that received the FDA’s *“Breakthrough” approval* designation for the treatment of patients with CLL. It also received the “Priority Review” designation. Obinutuzumab is intravenously administered in combination with oral chlorambucil for six 28-day cycles, with obinutuzumab given days 1, 8 and 15 of cycle 1 and then day 1 of cycles 2-6 in previously untreated CLL patients^26^. **

Given that the anti-CD20 antibody rituximab, first approved in 1997 for relapsed indolent lymphoma, has transformed the treatment of B cell diseases, many research strategies have focused on the development of novel anti-CD20 antibodies. Most of these have been engineered to enhance affinity for Fc receptors (CD16) to improve ADCC, though some are better at fixing complement. These have been type I antibodies that require translocation into lipid rafts. Obinutuzumab is a glyco-engineered antibody. However, its main difference is purported to be that it is a type II antibody that does not require lipid rafts and is more potent in terms of direct cytotoxicity^25^. Whether this is in fact true in the clinical situation remains unclear, since obinutuzumab was administered at a higher dose and more dose-dense schedule than rituximab in this study.

Obinutuzumab has several reported and potential side effects common to other infused monoclonal anitbodies, including the risk for infusion reactions which was higher than for rituximab and required glucocorticoid pre-medication, leucopenia (infections), thrombocytopenia (easy bruising, bleeding), anemia, Progressive Multifocal Leuko-encephalopathy (PML), Hepatitis B Virus (HBV) reactivation, fever, pain (muscle and joints) and cough^27^.

## Lenalidomide (Revlimid) - a novel FDA approved drug for the therapy of MCL (2013)

Lenalidomide is a novel drug produced by Celgene that was approved for treatment of patients with relapsed multiple myeloma in 2006 (in combination with dexamethasone) and that also received approval in February 2013 by the FDA for the new indication of treatment of relapsed/progressive MCL (FDA website) after two prior therapies^31^. MCL is a relatively aggressive type of B cell lymphoma, representing approximately 5-10% of the non-Hodgkin lymphomas^32^. Lenalidomide demonstrated an overall response rate of ~26% and a complete response in 7% in these heavily pre-treated patients. For the patients that responded to treatment, the median response duration was of ~ 16 months^31^.

Important side effects, as expected from its years of prior use, included: leucopenia/neutropenia (infections), thrombocytopenia (easy bruising, bleeding), anemia (fatigue), diarrhea/constipation, nausea, cough, fever, rash/pruritus, dyspnea, and peripheral edema. Due to serious adverse events, almost one-fifth of the patients had to discontinue the lenalidomide therapy. When combined with dexamethasone, deep venous thrombosis, intracranial thrombosis, and pulmonary embolism were reported in a small number of cases^31,33^.

Lenalidomide is considered an immuno-modulatory drug, synthesized by modifying thalidomide, though its precise mechansims of action remain unclear. The modifications resulted in increased potency and altered side effect profile compared to thalidomide^34^. In addition to its immunomodulatory effects (acting both on the cellular and humoral immunity), lenalidomide was shown to be also anti-angiogenic and to modify critical signaling pathways^34,35^. For example, lenalidomide is able to destabilize the p27/Cyclin D1 complexes during cell cycle progression and can inhibit the NF-kB pathway by a cereblon-dependent inhibition of IRF4 expression^36,37^.

## Conclusions

Innovations and new developments are continuously desired and needed in most, if not all, scientific fields and disciplines^38-42^. However, there is a specifically continuous and critical need for finding novel drug targets and for developing improved drugs, with greater potency and lower side effects, that will target currently incurable diseases, such as many hematologic malignancies. Critical unmet needs in lymphoid malignancies are to develop curative therapies for patients with aggressive subtypes that are not cured by initial therapy, and for all indolent subtypes.

As Garth L. Nicolson (Professor Emeritus, Institute for Molecular Medicine in Huntington Beach, California) said *“by working between different areas and incorporating information and techniques from different disciplines new progress and breakthroughs can be made on important problems”*^43^. Recent advances in genome technology and its use in the discovery of genetic basis of differences between tumors of same type in different patients is a big step forward. This is a promising approach that will result in targeted therapies, enhanced drug potencies, and potential decrease in side effects^44^.

According to American Cancer Society, cancer is the second cause of death in the USA, with one out of four deaths being due to a malignancy^45^. Overall, between 2006 and 2013, the trend of new FDA drug approvals and new drugs for cancer treatment remains relatively constant, with a peak in 2012, and a drop in 2013.

Ibrutinib, obinutuzumab, and lenalidomide represent the three FDA approved drugs in the past 15 months specifically used for the treatment of the B cell malignancies CLL and/or MCL. They have non-overlapping mechanisms of action and of toxicity, opening up numerous possibilities for combination therapy. We expect approval later in 2014 of another anti-CD20 antibody (ofatumomab)^46,47^, another B cell signaling inhibitor, the PKC-delta isoform inhibitor idelalisib^48,49^, and possibly a novel pro-apoptotic BCL-2 inhibitor (ABT-199)^50,51^. These new targeted agents likely will further transform the way these diseases are treated. A challenge for clinicians is to optimize their use, either alone and in rational combinations.

## KEY POINTS

- **Emergence of resistance and the lack of cures need to be addressed by:**
  - **rational development of combination therapies **
  - **development of** **novel drugs with enhanced potency to achieve better overall and complete response rates with decreased toxicity** **.**

- **Several important drugs were approved for the treatment of leukemia (CLL) and/or lymphoma (MCL), between January 2013 and March 2014:** **ibrutinib, obinutuzumab and lenalidomide**

- **Several expected FDA approvals: **
  - **anti-CD20 antibody ofatumomab**
  - **the PKC-delta isoform inhibitor *idelalisib*, and possibly **
  - a** pro-apoptotic Bcl-2 inhibitor *ABT-199*.**
